# Irreversible Electroporation (IRE) for Prostate Cancer (PCa) Treatment: The State of the Art

**DOI:** 10.3390/jpm14020137

**Published:** 2024-01-25

**Authors:** Eliodoro Faiella, Domiziana Santucci, Daniele Vertulli, Elva Vergantino, Federica Vaccarino, Gloria Perillo, Bruno Beomonte Zobel, Rosario Francesco Grasso

**Affiliations:** Department of Radiology, Policlinico “Campus Bio-medico”, Via Alvaro del Portillo, 200-00128 Rome, Italy; e.faiella@policlinicocampus.it (E.F.); d.santucci@policlinicocampus.it (D.S.); elva.vergantino@unicampus.it (E.V.); federica.vaccarino@unicampus.it (F.V.); gloria.perillo@unicampus.it (G.P.); b.zobel@policlinicocampus.it (B.B.Z.); r.grasso@policlinicocampus.it (R.F.G.)

**Keywords:** irreversible electroporation, prostate cancer, cancers treatments, interventional radiology, IRE

## Abstract

We evaluated the most recent research from 2000 to 2023 in order to deeply investigate the applications of PCa IRE, first exploring its usage with primary intent and then salvage intent. Finally, we discuss the differences with other focal PCa treatments. In the case of primary-intent IRE, the in-field recurrence is quite low (ranges from 0% to 33%). Urinary continence after the treatment remains high (>86%). Due to several different patients in the studies, the preserved potency varied quite a lot (59–100%). Regarding complications, the highest occurrence rates are for those of Grades I and II (20–77% and 0–29%, respectively). Grade III complications represent less than 7%. Regarding the specific oncological outcomes, both PCa-specific survival and overall survival are 100%. Metastasis-free survival is 99.6%. In a long-term study, the Kaplan–Meier FFS rates reported are 91% at 3 years, 84% at 5 years, and 69% at 8 years. In the single study with salvage-intent IRE, the in-field recurrence was 7%. Urinary continence was still high (93%), but preserved potency was significantly lower than primary-intent IRE patients (23%). In addition, Grade III complications were slightly higher (10.8%). In conclusion, in males with localized low–intermediate-risk prostate cancer, IRE had an excellent safety profile and might have positive results for sexual and urinary function.

## 1. Introduction

In the last decades, due to widespread screening practices, prostate cancer (PCa) has recently been identified at earlier stages. Although drastic procedures, such as radical prostatectomy (RP) and external beam radiation therapy (EBRT), are considered as the standard treatments for patients with low to intermediate risk [1], a currently growing interest about overtreatment has prompted researchers to work towards investigating less invasive alternatives. Partial prostate ablation, which sits in the midst of active surveillance and radical treatments, attempts to treat PCa while preserving the gland tissue portion necessary to maintain genitourinary function [2].

Multiparametric magnetic resonance imaging (mpMRI) has demonstrated to be extremely accurate both for the localization of PCa lesions and the guiding of target lesion biopsy [3,4]. The correct identification of a lesion is necessary before ablation treatment. Additionally, the creation of ablative technology has made it possible for us to remove tumor foci while protecting adjacent tissue.

Irreversible electroporation (IRE) stands at the forefront of emerging ablative techniques, and is currently under thorough investigation within the medical community. For urologists seeking to incorporate these novel technologies into their practice, a comprehensive understanding of the scientific foundations is imperative. Unlike conventional treatment modalities that rely on non-selective thermal destruction, IRE represents a paradigm shift by employing a non-thermal approach, strategically designed to annihilate targeted cells while preserving vital structures. The distinctive feature of IRE lies in its utilization of brief yet potent electrical fields, harnessing their capacity to puncture cell membranes permanently and lethally. This precise disruption of cellular membranes results in the creation of nanopores and a consequential modification of cell membrane permeability. The cumulative effect of these processes induces apoptosis, providing a unique mechanism for cell death that distinguishes IRE from other ablative techniques. It is noteworthy that despite being primarily characterized as a non-thermal mechanism, recent studies, particularly in porcine models, have elucidated the generation of heat during the IRE process. This intriguing revelation adds a layer of complexity to the understanding of IRE, suggesting that the interplay between non-thermal and thermal effects may contribute to the overall efficacy of the treatment. As researchers delve deeper into the intricacies of IRE, exploring its mechanisms at the cellular and molecular levels, the potential applications and nuances of this technology become increasingly apparent. The ability to selectively target cells for destruction without causing collateral damage to surrounding structures holds great promise for various medical fields, especially in the realm of urology. The ongoing exploration of IRE represents a pioneering venture into the realm of focused ablative techniques. Its non-thermal methodology, coupled with the recent insights into heat generation, adds a layer of sophistication to its application. Urologists and radiologists navigating this landscape of innovation must continue to unravel the complexities of IRE, paving the way for its judicious and effective integration into clinical practice [5].

Providing sharp ablation zone margins is one of IRE’s benefits. It is generally agreed that only a few cell layers separate the reversibly electroporated area from the irreversibly electroporated area. In contrast, there are no transition zones like in thermal- or radiation-based ablation procedures. Real-time monitoring is an additional advantage. Both during and after the treatment, it is possible to partially view the treatment volume. Possible visualization techniques include MRI, CT, and ultrasound [6].

In this review, we critically evaluate the most recent researches in order to deeply investigate the PCa IRE applications, first exploring its primary-intent usage and then its salvage-intent usage. Finally, we discuss the differences with other focal PCa treatments.

## 2. Materials and Methods

**Figure 1:**
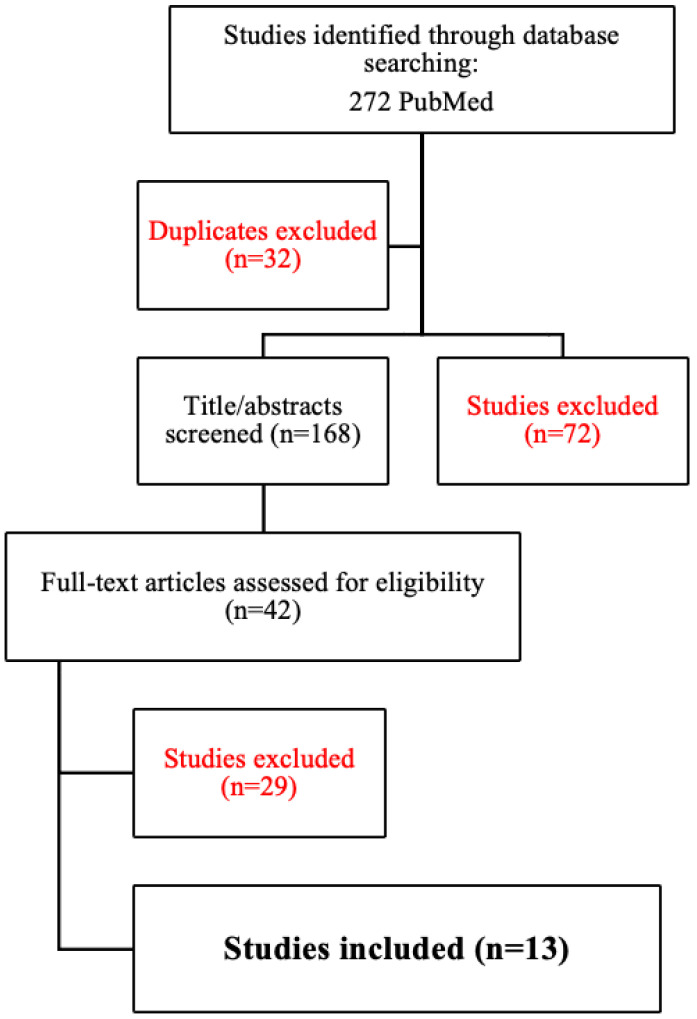
PRISMA (preferred reporting items for systematic reviews and meta-analysis) 2009 flowchart for study search and selection. Red color data have been excluded because of no utility for this study or other reasons.

A literature search was performed using the Medical Literature Analysis and Retrieval System Online (MEDLINE), the Excerpta Medica dataBASE (EMBASE), PubMed, and Google Scholar. The following terms were entered into the search algorithm to identify articles: ‘prostate irreversible electroporation’, ‘irreversible electroporation’. The search was limited to articles in English published between 2000 and 2023. The authors reviewed the retrieved articles by using a PRISMA flowchart (Figure 1), and the references of the retrieved articles were used when relevant.

## 3. Results

A total of 13 studies were included in this review, which were divided according to the modality of the IRE (primary-intent and salvage-intent after RT). Finally we included a single study which reported the differences between focal and diffuse IRE. In particular, 12 studies were in a primary-intent IRE setting, and 1 was in a salvage-intent setting.

See Table 1 for the complete overview of the studies.

### 3.1. Primary Intent

In a first study [7], 16 patients with ages ranging from 40 to 78 were treated by using IRE. All patients were continent, and those who were potent before the operation remained potent after it. For two individuals who had bilateral regions treated, it took six months for their full potency to return. In 15 individuals (94%), postoperative biopsies from the site of confirmed malignancy revealed no sign of the disease.

After 12 months to IRE, all 16 of the males in the study by Valerio et al. [20] had pad-free and leak-free continence. The number of males who had an erection strong enough to penetrate dropped from 75% to 69%. There were no significant adverse effects noted. Changes in EPIC (Expanded Prostate Cancer Index Composite) and I-PSS (International Prostate Symptom Score) showed a statistically significant improvement in urine symptoms (*p* = 0.039 and 0.001, respectively).

Analogous results were obtained in another study [9], where 34 patients were recruited. A total of 100% (24/24) of patients were continent, and 95% (19/20) maintained their potency.

Between these patients, most of them had low or moderate risk disease (26% and 71%, respectively). A total of 6 months after the treatment, there were 12 Grade I (35%) and 10 Grade II (29%) complications. No patient experienced a problem of Grade III or more. The authors concluded by saying that additional prospective development studies are required to investigate the oncological potential and confirm the functional outcomes.

In order to indagate oncological outcomes, the study by Ting et al. [10] comprised 25 patients with low–intermediate-risk PCa who had not previously had PCa therapy. A total of 4% Grade III complications and 20% Grade 1 complications were discovered during follow-up. According to previous literature, no discernible change in American Urological Association urine symptom score, sexual function, or bowel function was seen at functional follow-up. No unusual abnormalities in-field in any of the patients were found after the treatment. No in-field recurrences occurred, and after 8 months, 76% of patients were histologically free of substantial malignancy. The treatment margins were widened to account for the fact that almost all recurrences occurred close to the treatment zone.

Collettini et al. [14] reported that there was no statistical difference in the proportion of men with preserved potency and leak-free and pad-free continence rates. According to modifications in the International Consultation on Incontinence Questionnaire Male Lower Urinary Tract Symptoms (ICIQ-MLUTS) and the International Index of Erectile Function (IIEF-5) questionnaires, urogenital function remained steady at 12 months. Multiparametric prostate MRI and targeted biopsies performed at 6 months revealed that the percentage of in-field treatment failure was 17.9%.

Being more specific about IRE complications, Murray et al. [11] reported 2 (8%) Grade III complications, including epididymitis and urinary tract infection, and 14 Grade II or lower complications (58%), including transitory urine symptoms, hematuria, and urinary tract infections. A total of 16% of the 25 patients developed malignancy in the ablation zone during regular follow-up biopsy at 6 months. Of those who had normal urine function at baseline, 88% and 94% reported having normal urinary function at 6 and 12 months following prostate gland ablation, respectively.

Van der Bos et al. [13] performed IRE ablation on high-volume disease with any Gleason sum score of 7 (ISUP Grades II–III) or any Gleason sum score of 6 (ISUP Grade I), including 63 patients in total. There were no high-grade adverse events according to the previous literature mentioned above. There was a little decline in the sexual QoL dimension (66 at baseline vs. 54 at 6 months, *p* = 0.001), but there was no significant change in the physical, mental, intestinal, or urinary QoL domains from baseline. On follow-up biopsies, the in-field and whole-gland oncological control rates were 84% (38/45 patients) and 76% (34/45 patients), respectively. They tried to find a reason for oncological failure of IRE ablation, and they reported that system faults (*p* = 0.010) and a small safety margin (*p* = 0.047) were shown to be possible early risk factors.

A recent study [16] valuated the IRE ablation of apical PCa lesions, including 50 patients who had a PCa lesion within 3 mm. In the EPIC urine or bowel QoL domain, there was no discernible difference between the baseline and 12-month post-treatment periods. A total of 12 months after therapy, one patient (2%) needed one pad per day to manage urine incontinence. EPIC sexual QoL decreased but not in a significantly way (from 65 at baseline to 59 at 12 months post-IRE). A total of 94% of the patients’ pre-treatment potency persisted following therapy. Only one patient (2.5%) exhibited in-field residual disease.

Another study [15] analyzed 123 IRE-treated patients; of them, 76% exhibited no change in erectile function, and 98.8% continued to be pad-free. In-field recurrence was found in 2.7–9.8% of patients. Regarding FFS at three years, metastasis-free survival at that time was 99% and overall survival was 100%. A total of 18 patients required salvage therapy (12 underwent repeat IRE, and 6 underwent whole-gland therapy). Their conclusion was that focal IRE has acceptable short-term oncological results with little effect on patient quality of life in a subset of patients with localized clinically significant PCa.

A recent study [19] aimed to report both oncological and functional outcomes of IRE as the primary therapy for 223 locally advanced clinically significant PCa at a median follow-up of 5 years (up to 10 years). Short-term urine continence was maintained (98–99%); however, the number of erections strong enough for sexual activity fell by 13% from baseline (71% to 58%). Kaplan–Meier FFS rates were 91% at 3 years, 84% at 5 years, and 69% at 8 years. PCa-specific and overall survival were both 100%, whereas metastasis-free survival was 99.6%. Follow-up biopsy revealed residual PCa in 24% (45/190) of the cases. In this unique single-center longer-term follow-up, focused IRE showed acceptable local and distant oncological outcomes in a subset of males with less toxicity to the urinary system and sexual organs than radical therapy.

### 3.2. Salvage Intent after RT

In a single-center study [18], 74 males with biopsy-proven radio-recurrent PCa were treated with IRE. At the 12-month follow-up, 93% of the patients had maintained urine continence, and 23% had maintained erectile function. A total of 57 patients (77%) had local control and required no additional treatment. In-field recurrence occurred in 7% of patients, while out-field recurrence occurred in 15% of patients. A total of 91% of patients survived without developing metastases, with a median duration to metastases of 8 months. A sum of 60% was the predicted Kaplan–Meier 5-year progression-free survival rate. They came to the conclusion that these short- to mid-term safety, oncological, and quality-of-life outcome data support findings demonstrating the capacity of salvage-focused IRE to safely achieve oncological control in patients with radio-recurrent PCa.

### 3.3. Focal or Diffuse IRE?

In a study by de La Rosette et al. [17], in an investigation aimed at discerning the optimal approach for men with locally advanced, low-risk PCa, a meticulous randomization process was employed. This process led to the allocation of 51 patients to receive focused IRE ablation and 55 patients to undergo diffuse IRE. Quality-of-life assessments, utilizing measures such as IIEF-5, EPIC, and I-PSS, provided valuable insights into the comparative outcomes of these two distinct approaches.

Upon scrutinizing the data at the three-month follow-up, it was observed that rates of erectile dysfunction and adverse events were comparable between the two groups. Interestingly, the focal ablation group exhibited higher IIEF-5 scores, hinting at potential advantages in terms of erectile function within the initial three months. Notably, this observation was further supported by superior EPIC-sexual life scores in the focal ablation group compared to the extended ablation group.

However, it is crucial to highlight that other quality-of-life metrics did not show significant differences between the two groups, emphasizing the nuanced nature of the outcomes. The complexity of these results suggests that the impact of the ablation approach on various aspects of patients’ lives is multifaceted and requires comprehensive evaluation.

A pivotal aspect of the study involved a 6-month prostate biopsy, a critical milestone in assessing the efficacy of the two ablation strategies. Surprisingly, no substantial differences were identified between the focal and prolonged IRE groups in terms of residual clinically relevant PCa (Gleason > 3 + 4), with rates standing at 18.8% and 13.2%, respectively.

In the conclusive remarks, the researchers asserted that among males with localized low–intermediate-risk PCa, both focal and diffuse IRE ablation exhibited comparable safety profiles, urinary function, and oncologic outcomes. The key differentiator emerged in the early phases of treatment, where focused ablation demonstrated superior outcomes in erectile function compared to diffuse ablation during the initial 3–6 months. These findings underscore the importance of considering not only long-term outcomes but also the early and nuanced impact of treatment choices on patients’ quality of life.

## 4. Discussion

In order to prevent contractions during the IRE operation, a muscle relaxant agent is also administered to the patients under general anesthesia. A Foley catheter is placed to empty the bladder while the patient is in the lithotomy position, and it is maintained for at least two days following the treatment. The IRE console is composed by different monopolar needle electrodes and a direct current generator, and it is supervised by computer-based treatment planning software. Based on mpMRI and template-guided mapping prostate biopsies, preoperative targeted sites for ablation are identified [21].

The electrodes are positioned and guided in the predetermined targeted area using a trans-perineal template grid used for brachytherapy seed placement under ultrasound guidance. Following electrode insertion, IRE parameters, such as electrode exposure and distances between electrodes, are entered into the IRE system’s planning software before IRE execution. The whole treatment takes around an hour.

To obtain a current flow of 20–50 A between each electrode pair, the IRE technique contain 90 pulses, each lasting 70 msec. The electrodes are carefully removed after the energy transfer, leaving the urine catheter in situ. Depending on the size of the ablation zone and its proximity to the intraprostatic urethra, the urine catheter is withdrawn 2–10 days following the treatment.

Collettini et al., in their study [14], performed a transrectal contrast-material-enhanced US of the prostate utilizing the MRI-transrectal/US-fusion method the day before and the day after IRE in order to evaluate the perfusion changes within the predefined target volume during all the procedure.

Neal et al. [8] administered prostate IRE protocols to two human malignant prostates and a healthy canine prostate while monitoring electrical data. With a treatment volume that demonstrates fast lesion development and resolution, there is a sub-millimeter boundary between damaged and unaffected cells that is tunable based on electrode configurations and pulse settings. Treatment planning is made by the correlation between the destroyed volume and the distribution of the electric field, and real-time treatment monitoring is made by alterations in tissue properties. The electroporated tissue may trigger a supplemental immunological response. Crucially, the extracellular matrix is unaffected by the pulses, protecting delicate anatomical regions such the ductal structures, neurovascular bundles, and main arteries. Because of this, locations that are too risky for resection or other focused procedures can be treated with IRE with a low risk of morbidity.

In tiny animals, IRE has showed its usefulness in treating complicated malignancies in delicate areas. These include a brain tumor and a sizable sarcoma that covered the sciatic nerve and important blood arteries. As looked at in the short- and medium-term, IRE safely ablated both malignant human prostates and healthy canine prostates. In the human cases, the histological results reveal instances of full tissue necrosis in the center with varying tissue damage outside the periphery.

Onik et al. [7] used IRE in sixteen individuals between the ages of 40 and 78. Every patient was treated as an outpatient and had good tolerance for the treatment. The treated area had variable echogenicity, according to US collected during the surgery. When the prostatic capsule was included in the formed lesion, it was seen to be indistinct. Immediately following the surgery, color Doppler ultrasonography revealed unbroken flow inside the neurovascular bundle. There was a range of 0 to 3 days in the catheter drainage period. The patient who underwent treatment for lesions on both sides of the gland experienced the greatest duration. After the treatment, every patient who was potent prior to it became potent again. All patients were continent immediately following treatment.

The biopsy specimens obtained after IRE whole-gland treatment showed no signs of any functional glandular tissue. There were occasionally parenchymal areas with the ghostly remains of glandular structures; however, there was no presence of living cells. Intact nerve bundles and living ganglion cells were observed, encircled by fibrotic and necrotic tissue. Vascular components were also present and unobstructed.

In their study, van den Bos et al. proposed radical prostatectomy as scheduled to take place four weeks after IRE on 16 individuals with PCa [13]. The macroscopic examination discerned a distinct ellipsoid-shaped hemorrhagic region enveloping petite pale discolored zones situated at the center of the IRE ablation zone. Notably, the ablation volumes, exhibiting well-defined demarcation, spanned a spectrum from 5 to 40% of the prostate, extending comprehensively from the capsule to the prostatic urethra. It is noteworthy that one prostate specimen did not manifest any macroscopically assessable ablation zone, and the tracts of the electrodes were indiscernible. Upon microscopic scrutiny of the ablation zone, it was elucidated that fibrosis, necrosis, and ghost-tubuli with eosinophilic cytoplasm were discernible in 15 out of 16 patients. This region was encapsulated by a hemorrhagic area, corresponding with the precise location of the electrodes as identified through ultrasound imaging. Importantly, the ablation zone exhibited a well-demarcated nature, featuring a sharply defined boundary that delineated the viable and non-viable tissue zones. A singular prostate specimen solely exhibited fibrosis without a necrotic component. Additionally, the ablated tissue showcased mild to moderate inflammation across all cases, with one case displaying a focal severe inflammatory component characterized by atrophic cells. Glandular hyperplasia was evident in 11 prostates, and further histological analysis identified basal cell hyperplasia and transitional cell metaplasia in 10 and 1 cases, respectively. Importantly, no skip lesions, indicative of the absence of viable tissue, were observed within the ablated area. Pertinently, the IRE treatment impacted the prostate capsule in 12 out of 16 cases, showcasing invasion by adipocytes and lipophages within the capsule structure. The boundary between viable and nonviable tissue in the ablation zone was sharply defined. Mild to severe inflammation and, in one instance, atrophic cells were seen in the ablated tissue. At the site of the electrodes, there was bleeding all around the region. Thirteen individuals had fibrinoid necrosis of the neurovascular bundle, and nine had denudation of the urothelium of the prostatic urethra.

In primary-intent IRE, the in-field recurrence is quite low (ranges from 0% to 33%). Urinary continence after the treatment remains high (>86%). Due to several different patients in the studies, the preserved potency varied by quite a lot (59–100%). Regarding complications, the highest are Grades I and II (20–77% and 0–29%, respectively). Grade III complications are less than 7%. About the specific oncological outcomes, both PCa-specific survival and overall survival is 100%. Metastasis-free survival is 99.6%. In a long-term study, the Kaplan–Meier FFS rates reported are 91% at 3 years, 84% at 5 years, and 69% at 8 years.

In the single study with salvage-intent IRE, the in-field recurrence was 7%. The urinary continence was still high (93%), but preserved potency was significantly lower than primary-intent IRE patients (23%). In addition, Grade III complications were slightly higher (10.8%).

Scheltema et al. [12] compared clinical outcomes of IRE patients vs. radical prostatectomy patients. In terms of maintaining pad-free continence and erections strong enough for intercourse, IRE was noticeably superior to radical prostatectomy. There were no statistically significant differences between the EPIC summary scores. Grade I complications were 22% and 16% for IRE and RP, respectively; Grade II complications were 13.7% and 9% for IRE and RP, respectively; there were no Grade III complications for either IRE or RP. Despite IRE patients initially complaining more, urinary symptoms for both groups of patients were improved after 12 months. A limitation is that individuals with IRE had more early oncological failure than individuals with radical prostatectomy.

In the comprehensive examination of treatments, a detailed comparative review [22] between Irreversible Electroporation (IRE) and High-Intensity Focused Ultrasound (HIFU) has unveiled intriguing insights into their respective outcomes. Patients undergoing IRE exhibited notable advantages, including higher mean rates of in-field negative post-treatment biopsy results, lower mean prostate-specific antigen (PSA) level decreases, and superior rates of potency maintenance compared to their HIFU counterparts. The nuanced analysis of adverse events further enriched the comparison. Commonly reported issues, such as urinary tract infection, dysuria, hematuria, and incontinence or urgency, were prevalent in both IRE and HIFU treatments. Remarkably, the majority of these adverse events were categorized as equivalent and mild, falling within Grades I or II. This underscores the overall safety profile of both treatments, emphasizing the manageable nature of the reported side effects. Delving deeper into the safety assessment, it is noteworthy that after IRE and HIFU procedures, only a modest percentage of patients, ranging from 0 to 8%, experienced serious adverse events categorized as Grade III. This low incidence of severe complications suggests a favorable risk profile for both IRE and HIFU, highlighting their suitability for individuals seeking minimally invasive treatment options for prostate-related conditions. Equally important, both IRE and HIFU demonstrated commendable functional results and showcased their ability to preserve the quality of life (QoL) for treated individuals. This positive outcome is crucial in the evaluation of treatment modalities, as it indicates not only the therapeutic efficacy but also the holistic impact on patients’ well-being. As the scientific community continues to scrutinize the intricacies of IRE and HIFU, this comparative analysis serves as a valuable reference for clinicians and researchers alike. The ongoing pursuit of understanding the nuanced differences and similarities between these innovative treatment approaches will undoubtedly contribute to the refinement and optimization of prostate care, ultimately offering patients a spectrum of choices tailored to their individual needs and preferences.

## 5. Conclusions

In males diagnosed with localized low–intermediate-risk prostate cancer, IRE has demonstrated an exceptional safety profile and the potential for favorable outcomes in terms of both sexual and urinary function. The short-term oncological results of IRE are encouraging, suggesting its viability as a therapeutic option. Particularly noteworthy is the positive impact of IRE on patients with unifocal localized clinically significant PCa. This advanced technology may offer a pathway for these patients, allowing over 80% of them to sidestep more drastic therapeutic interventions even after a 5-year follow-up period. The appeal of IRE is evident, especially among individuals with screen-detected cancer who possess unifocal, favorable, low-volume, intermediate-risk disease. For many, IRE could potentially become the preferred initial course of treatment. Despite its promising prospects, widespread accessibility to IRE remains limited, currently confined to clinical trials and a select number of institutions. The potential benefits of this innovative treatment option are thus not yet available to the broader population. A future scenario where IRE is widely accessible could significantly alter the treatment landscape for men facing screen-detected cancer with specific disease characteristics. For the moment, the utilization of IRE hinges on more extensive multi-center trials and longer-term follow-up studies. These endeavors are crucial in establishing the broader effectiveness, safety, and sustained outcomes of IRE in the management of PCa. As this technology progresses through rigorous testing and evaluation, it holds the promise of becoming a mainstream and transformative therapeutic approach for a significant subset of PCa patients.

## Figures and Tables

**Table 1:** Overview of characteristics, outcomes, and complications of studies about IRE treatment.

Year	First Author	Intent	Patients	PSA (Mean), Age (Mean)	Outcomes	Complications
2010	Onik [7]	Primary	16	3–7; 40–78	No in-field recurrence 100% continence 100% preserved potency	Not reported
2014	Neal [8]	Primary	2	4.5; 61	No in-field recurrence	Not reported
2014	Valerio [9]	Primary	19	7.5; 60	In-field recurrence: 33% 100% continence 95% preserved potency	Grade I: 12 (35%) Grade II: 10 (29%) Grade III: 0
2016	Ting [10]	Primary	32	6; 67	No in-field recurrence 100% continence 100% preserved potency	Grade I: 5 (20%) Grade II: 0 Grade III: 1 (4%)
2016	Murray [11]	Primary	25	4.5; 63.2	In-field recurrence: 16% 100% continence 95% preserved potency	Grade I: 6 (22%) Grade II: 7 (29%) Grade III: 1 (7%)
2018	Scheltema [12]	Primary	50	5.9; 67	In-field recurrence: 29.5% 98% continence (same in RP) 69% preserved potency (68% in RP)	Grade… IRE vs. RP Grade I: 11 (22) vs. 9 (16%) Grade II: 7 (13.7%) vs. 5 (9%) Grade III: 0 vs. 0
2018	Van den Bos [13]	Primary	63	6; 67	In-field recurrence: 16% 100% continence 77% preserved potency	Grade I: 15 (24%) Grade II: 7 (11%) Grade III: 0
2019	Collettini [14]	Primary	30	8.65; 65.5	In-field recurrence: 17.9% 86.2% continence (vs. 90% at baseline) 79.3% preserved potency (vs. 83.3% at baseline)	Grade I: 6 (20%) Grade II: 3 (10%) Grade III: 1 (3.3%)
2020	Blazevski [15]	Primary	123	5.7; 68	In-field recurrence: 2.7–9.8% 98.8% continence 76% preserved potency FFS at three years: 99% Overall survival: 100%	Not reported
2021	Blazevski [16]	Primary	50	6.25; 68	In-field recurrence: 2.5% 98% continence 59% preserved potency (vs. 65% at baseline)	Grade I: 10 (20%) Grade II: 9 (18%) Grade III: 0
2022	De la Rosette [17]	Primary (local vs. diffuse IRE)	51	5.93; 64	Higher IEEF-5 and EPIC scores at 3 months follow up in focal IRE than diffuse IRE 6 month biopsy with residual PCa: 18.8% vs. 13.2% (no significance)	Grades… focal vs. diffuse Grade I: 23 (77%) vs. 27 (79%) Grade II: 6 (20%) vs. 7 (21%) Grade III: 9 (3.3%) vs. 0
2023	Geboers [18]	Salvage	74	5.4; 69	In-field recurrence: 7% 93% continence 23% preserved potency	Grade I-II: 6 (8.1%) Grade III: 8 (10.8%)
2023	Scheltema [19]	Primary	229	5.9; 68	In-field recurrence: 24% 98–99% continence 71% preserved potency (vs. 58% at baseline) Kaplan–Meier FFS rates: 3 y: 91% 5 y: 84% 8 y: 69% PCa-specific survival: 100% Overall survival: 100% Metastasis-free survival: 99.6%	Not reported

## Data Availability

Data is contained within the article.

## Abbreviations

EMBASE	Excerpta Medica dataBASE
EBRT	External beam radiation therapy
EPIC	Expanded Prostate Cancer Index Composite
HIFU	High-intensity focused ultrasound
ICIQ-MLUTS	International Consultation on Incontinence Questionnaire Male Lower Urinary Tract Symptoms
IIEF-5	International Index of Erectile Function
I-PSS	International Prostate Symptom Score
IRE	Irreversible electroporation
ISUP	Internetional Society of Urologist pathologists
MEDLINE	Medical Literature Analysis and Retrieval System Online
mpMRI	Multiparametric magnetic resonance imaging
PCa	Prostate Cancer
QoL	Quality of life
RP	Radical prostatectomy
RT	Radiotherapy
TRUS	Trans-rectal ultrasound
